# Diffuse midline glioma, H3K27-altered, mimicking central neurocytoma on MRI: A case report

**DOI:** 10.1016/j.radcr.2025.09.074

**Published:** 2025-10-28

**Authors:** Yasuyuki Kojita, Tomonori Kanda, Shiho Yokoo, Kazuki Yasuda, Tokinosuke Miyazaki, Feiya Zeng, Naoe Jimbo, Takashi Sasayama, Takamichi Murakami

**Affiliations:** a Department of Radiology, Kobe Graduate University School of Medicine, 7-5-2 Kusunoki-cho, Chuo-ku, Kobe, Hyogo 650-0017, Japan; b Department of Diagnostic Pathology, Kobe Graduate University School of Medicine, 7-5-2 Kusunoki-cho, Chuo-ku, Kobe, Hyogo 650-0017, Japan; c Department of Neurosurgery, Kobe Graduate University School of Medicine, 7-5-2 Kusunoki-cho, Chuo-ku, Kobe, Hyogo 650-0017, Japan

**Keywords:** Diffuse midline glioma, H3K27-altered, Central neurocytoma, Soap-bubble appearance, Magnetic resonance imaging

## Abstract

Diffuse midline glioma (DMG), H3K27-altered, is an aggressive central nervous system tumor that usually arises in midline structures such as the pons, thalamus, or spinal cord. We present a rare case of intraventricular DMG, H3K27-altered in a 35-year-old woman. MRI showed a soap-bubble appearance, initially suggestive of central neurocytoma, but histopathological and immunohistochemical analyses confirmed the final diagnosis of DMG, H3K27-altered. This is the first reported intraventricular DMG, H3K27-altered with a soap-bubble appearance, underscoring the importance of including this tumor in the differential diagnosis of intraventricular masses.

## Introduction

Diffuse midline glioma (DMG), H3K27M-mutant, was newly defined in the 2016 WHO Classification as an infiltrative central nervous system (CNS) glioma that predominantly affects children and adolescents [1]. In the 2021 WHO Classification update, additional subtypes involving alternative mechanisms of H3K27 methylation loss (such as EGFR mutation or EZHIP overexpression) were included, and the terminology was changed from “H3K27M-mutant” to “H3K27-altered” [2]. Regardless of histological appearance, this tumor is classified as CNS WHO Grade 4 and carries an extremely poor prognosis, with a 5-year survival rate reported to be less than 1% [3,4]. These gliomas typically arise in midline structures of the CNS such as the pons, thalamus, or spinal cord, although rare cases with lesions involving the ventricular system have been reported [[5], [6], [7], [8], [9], [10], [11], [12], [13]]. However, due to their rarity, the imaging characteristics of intraventricular cases have not been fully elucidated.

Central neurocytoma is a rare intraventricular tumor classified as a CNS WHO Grade 2 neuronal neoplasm, typically arising in the lateral ventricles near the foramen of Monro [14]. It accounts for approximately 0.25–0.5% of all intracranial tumors, generally exhibits benign biological behavior, and is associated with a favorable prognosis when gross total resection is achieved [15]. On MRI, the presence of multiple intratumoral cysts, known as the soap-bubble appearance, is recognized as a characteristic feature [15].

We report an extremely rare case of a DMG, H3K27-altered, that presented with a soap-bubble appearance and mimicked a central neurocytoma on MRI.

## Case presentation

**Fig. 1:**
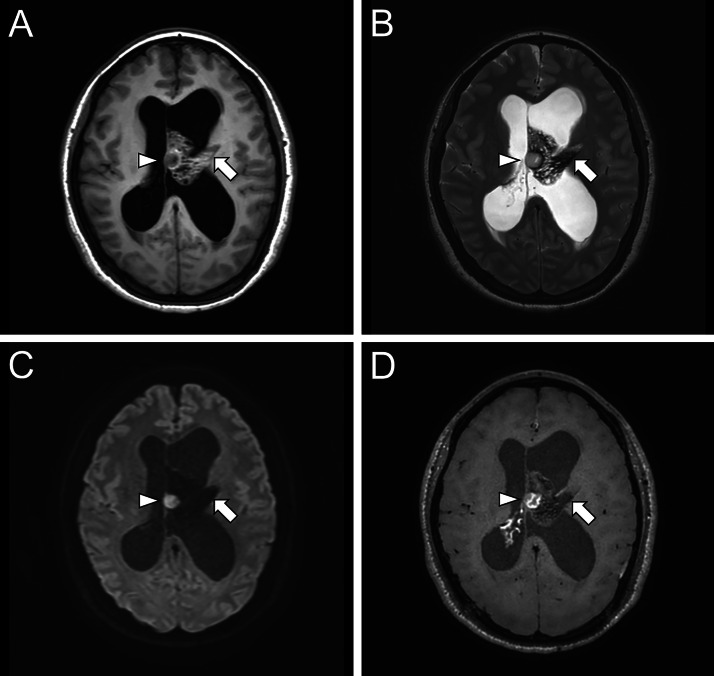
Initial brain MRI. A tumor is seen in the left lateral ventricle, wedge-shaped and continuous with the superior thalamus (arrow), causing hydrocephalus. It appears isointense to the surrounding white matter on T1- weighted imaging (A), and hypointense with internal cysts suggesting a soap-bubble appearance on T2-weighted imaging (B). Diffusion-weighted imaging (b = 1000 s/mm²) (C) and reveals nodular high signal, corresponding to contrast enhancement on postcontrast T1-weighted imaging (D) (arrowhead).

A 35-year-old female presented with a 2-week history of headaches. She had a history of uterine fibroids but no other relevant medical conditions. Her family history was negative for brain tumors or neurological diseases. Blood tests showed no remarkable abnormalities. Initial brain MRI (Fig. 1) revealed a tumor in the left lateral ventricle, resulting in obstructive hydrocephalus. The tumor was predominantly cystic and showed a wedge-shaped configuration abutting the left thalamus. The cystic components had signal characteristics similar to cerebrospinal fluid, consistent with the soap-bubble appearance. A portion of the lesion exhibited nodular restricted diffusion (minimum ADC value: 596 × 10⁻³ mm²/s), corresponding to contrast enhancement on postcontrast T1-weighted imaging. In addition, a solid component with contrast enhancement was observed extending into the third ventricle (not shown).

A craniotomy and partial resection of the tumor were performed at another hospital, but a definitive pathological diagnosis could not be made at that time. The patient’s symptoms initially improved; however, 2 months later she developed recurrent headaches and nausea. Imaging revealed enlargement of the residual tumor and the appearance of a new lesion in the fourth ventricle, consistent with tumor dissemination, along with worsening hydrocephalus. The patient underwent intensity-modulated radiotherapy (54 Gy in 30 fractions) and chemotherapy with cisplatin, cyclophosphamide, and vincristine, which resulted in shrinkage of the fourth ventricular lesion and reduced enhancement of the lesions in the left lateral and third ventricles. Nevertheless, new lesions subsequently appeared and grew around the left lateral ventricle, leading to the patient’s admission to our hospital for surgery 14 months after the initial presentation.

**Fig. 2:**
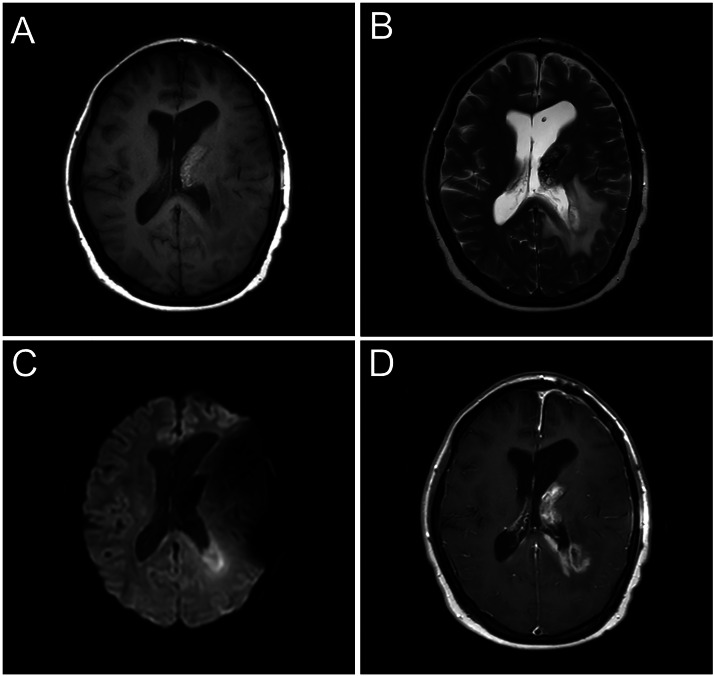
Brain MRI 14 months after initial presentation. The residual tumor in the left lateral ventricle appears hyperintense on T1-weighted imaging (A) and hypointense on T2-weighted imaging (B) relative to white matter. Diffusion-weighted imaging (b = 1000 s/mm²) (C) shows a newly emerged area of high signal around the trigone of the left lateral ventricle. Postcontrast T1-weighted imaging and (D) reveals contrast enhancement in both the residual lesion and the peritrigonal region of the left lateral ventricle.

Preoperative MRI at our hospital (Fig. 2) showed residual intraventricular tumor. The lesion, which was predominantly cystic and adjacent to the left thalamus, demonstrated heterogeneous enhancement. Around the left lateral ventricle, a new area exhibited restricted diffusion (minimum ADC value: 518 × 10^−3 mm²/s) and heterogeneous enhancement, consistent with a disseminated lesion.

**Fig. 3:**
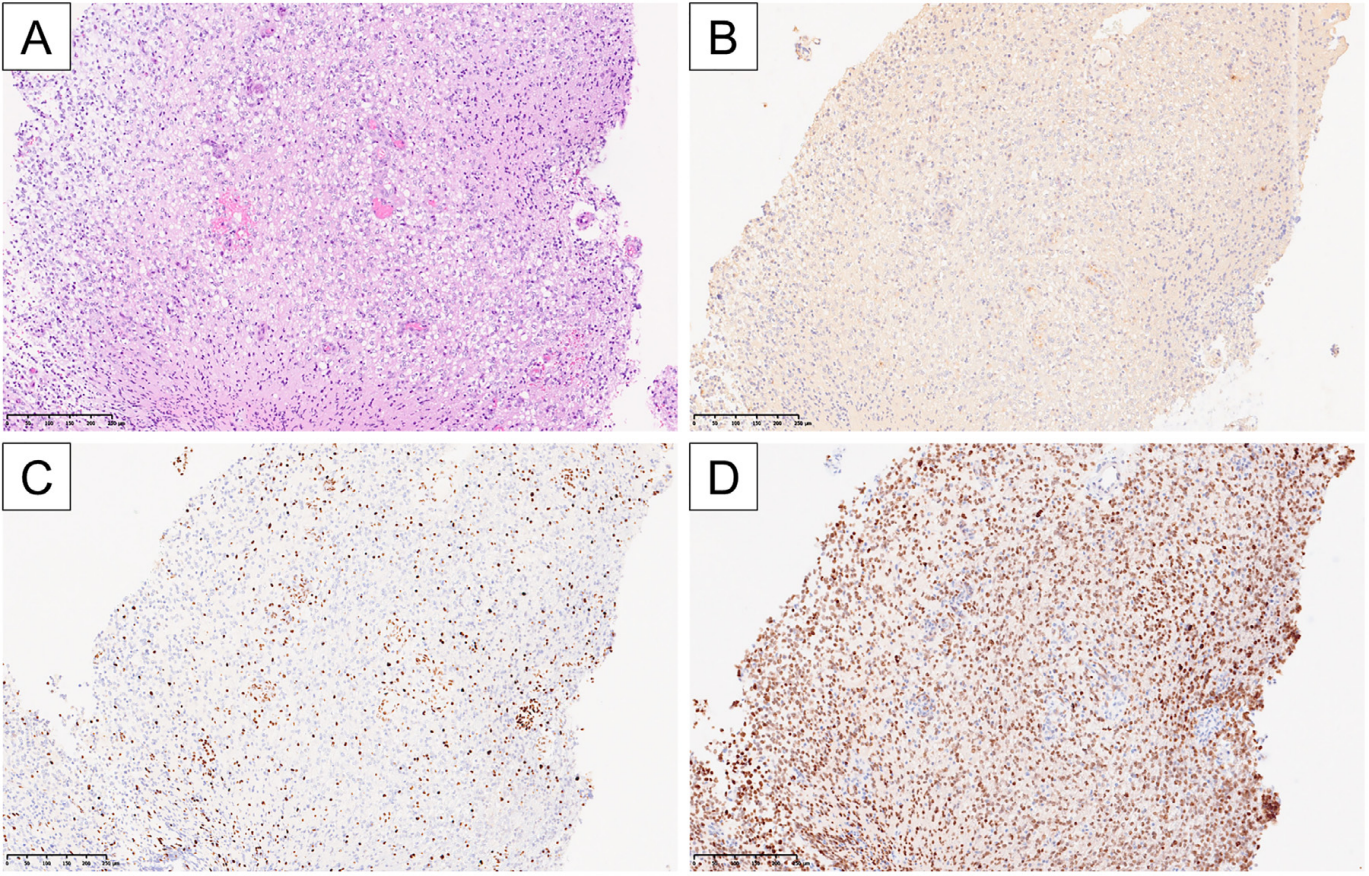
Histopathological findings. Hematoxylin and eosin (H&E) staining (A; 10×) shows a proliferation of cells with astrocytic morphology, consistent with an infiltrative glioma. No necrosis is observed, but microvascular proliferation is present. Immunohistochemically, mutant IDH1(R132H) is negative (B), H3K27me3 staining shows loss of expression in the tumor cells with positive internal control in endothelial cells (C), and H3K27M is positive (D).

A second craniotomy was performed, and the tumor was again partially resected. Histopathological examination (Fig. 3) showed a proliferation of cells with astrocytic morphology on hematoxylin and eosin (H&E) staining, consistent with an infiltrative glioma. No necrosis was observed, but microvascular proliferation was present. Immunohistochemistry revealed a complete loss of p53 expression and a Ki-67 labeling index of 30%. The tumor was positive for Olig2, negative for BRAF V600E, and negative for mutant IDH1 (R132H). Trimethylated H3K27 (H3K27me3) immunostaining showed loss of expression in the tumor cells with positive internal control in the endothelial cells, and H3K27M immunostaining was positive. These findings led to a final diagnosis of DMG, H3K27-altered.

After the diagnosis, the patient underwent stereotactic radiotherapy (25 Gy in 5 fractions) and chemotherapy with temozolomide and bevacizumab. Despite additional radiotherapy and chemotherapy, her condition gradually worsened with disorientation and apraxia, and she died 26 months after the initial presentation.

## Discussion

We reported a case of a DMG, H3K27-altered that presented as an intraventricular mass and was initially difficult to distinguish from a central neurocytoma. This tumor type is characterized by a point mutation in the histone H3 gene that substitutes lysine (K) 27 with methionine (M), resulting in hypomethylation in the histone tail and disruption of the polycomb repressive complex function [16]. This mutation occurs primarily in the H3F3A gene (approximately 70% of cases) but can also involve HIST1H3B or HIST1H3C [14,17,18]. Owing to this mutation, the tumor exhibits high-grade biological behavior even in the absence of classic high-grade histological features (such as high mitotic activity or necrosis).

The initial MRI of our case showed a mass extending from the lateral to the third ventricle with a soap-bubble appearance, making it difficult to differentiate from a central neurocytoma, a neuronal tumor (CNS WHO Grade 2) with relatively benign behavior and an excellent prognosis if gross total resection is achieved. The soap-bubble appearance refers to the presence of numerous variably sized cysts within a tumor, giving the impression of soap bubbles on imaging, and is widely recognized as a characteristic MRI feature of central neurocytoma. Although this feature is highly sensitive for central neurocytoma, its specificity is only about 58%, so careful differentiation from other intraventricular tumors is important [15]. The tumor in our case showed a wedge-shaped contour abutting the left thalamus, which differed from the undulating ventricular margin known as the scalloping sign [15] characteristic of central neurocytoma. This difference may have been helpful in distinguishing our lesion from a central neurocytoma.

To our knowledge, this is the first reported case of an intraventricular DMG, H3K27-altered with a soap-bubble appearance. In general, DMG, H3K27-altered, arises in midline structures such as the pons, thalamus, or spinal cord; intraventricular involvement is rare. Table 1 summarizes 27 reported cases of DMG involving the ventricular system. The median age was 25 years, with a slight male predominance (15 males, 12 females). Lateral ventricle involvement was the most common, but lesions in the third or fourth ventricle were also reported. Imaging findings were diverse and nonspecific, and no previous reports have described a soap-bubble appearance as observed in our case. On MRI, lesions were typically iso- to hypointense on T1-weighted images and hyperintense on T2-weighted images, and could exhibit restricted diffusion or intratumoral hemorrhage. Some cases demonstrated ring enhancement, but the degree of enhancement varied. Notably, most reported cases have come from Asia, suggesting the possibility of a genetic predisposition or a publication bias.

**Table 1:** Summary of reported cases of H3 K27-altered diffuse midline gliomas involving the ventricular system.

Reference	Age (years)	Gender	No. of cases	Ventricular location	Region
Luo et al. [5]	38	Male	1	Lateral ventricle	China
Wang et al. [6]	Median 13 (Range 11-59)	1 male and 2 female	3	Lateral (*n* = 2), Fourth (*n* = 1)	China
Wang et al. [7]	54	Female	1	Third ventricle	China
Zhao et al. [8]	14	Female	1	Lateral ventricle	China
Zheng et al. [9]	Median 24 (Range 7-65)	10 male and 6 female	16	Not specified	China
Matsunaga K et al. [10]	19	Female	1	Lateral ventricle	Japan
PL Kwok et al. [11]	41	Male	1	Lateral ventricle	Hong Kong
Jasso M et al. [12]	24	Male	1	Lateral ventricle	USA
Mitchel H et al. [13]	30	Male	1	Third ventricle	USA
Presenting case	35	Female	1	Third ventricle and lateral ventricle	Japan

Because central neurocytoma and DMG, H3K27-altered differ greatly in treatment strategies and prognosis, distinguishing between the 2 is crucial. Central neurocytoma has a favorable prognosis if gross total resection is achieved. In contrast, there is no curative therapy for DMG, H3K27-altered, and treatment is mainly aimed at prolonging survival and alleviating symptoms [16,19,20]. Typically, management includes radiation therapy combined with chemotherapy (such as temozolomide and bevacizumab), and surgical resection if the tumor’s location makes it feasible. However, clear treatment guidelines are lacking, and decisions must be individualized for each case. Therefore, when encountering an intraventricular tumor with a soap-bubble appearance, not only central neurocytoma but also DMG, H3K27-altered should be considered in the differential diagnosis.

## Conclusion

In conclusion, a case of a DMG, H3K27-altered, occurring in an adult female’s ventricular system that mimicked a central neurocytoma on MRI was presented. The prognoses of these 2 tumors differ significantly, so an accurate diagnosis is crucial for determining the appropriate treatment strategy. Continued accumulation of radiologic and clinical observations will help improve diagnostic accuracy for this rare tumor.

## Declaration of generative AI and AI-assisted technologies in the writing process

During the preparation of this work the authors used ChatGPT (https://openai.com/chatgpt) in order to improve readability and language. After using this tool, the authors reviewed and edited the content as needed and take full responsibility for the content of the publication.

## Patient consent

Written, informed consent was obtained from the patient for publication of this case.
